# Understanding the Community Risk Perceptions of the COVID-19 Outbreak in South Korea: Infodemiology Study

**DOI:** 10.2196/19788

**Published:** 2020-09-29

**Authors:** Atina Husnayain, Eunha Shim, Anis Fuad, Emily Chia-Yu Su

**Affiliations:** 1 Graduate Institute of Biomedical Informatics College of Medical Science and Technology Taipei Medical University Taipei Taiwan; 2 Department of Biostatistics, Epidemiology, and Population Health Faculty of Medicine, Public Health and Nursing Universitas Gadjah Mada Yogyakarta Indonesia; 3 Department of Mathematics Soongsil University Seoul Republic of Korea; 4 Clinical Big Data Research Center Taipei Medical University Hospital Taipei Taiwan; 5 Research Center for Artificial Intelligence in Medicine Taipei Medical University Taipei Taiwan

**Keywords:** Google Trends, risk, perception, communication, COVID-19, South Korea, outbreak, infodemiology

## Abstract

**Background:**

South Korea is among the best-performing countries in tackling the coronavirus pandemic by using mass drive-through testing, face mask use, and extensive social distancing. However, understanding the patterns of risk perception could also facilitate effective risk communication to minimize the impacts of disease spread during this crisis.

**Objective:**

We attempt to explore patterns of community health risk perceptions of COVID-19 in South Korea using internet search data.

**Methods:**

Google Trends (GT) and NAVER relative search volumes (RSVs) data were collected using COVID-19–related terms in the Korean language and were retrieved according to time, gender, age groups, types of device, and location. Online queries were compared to the number of daily new COVID-19 cases and tests reported in the Kaggle open-access data set for the time period of December 5, 2019, to May 31, 2020. Time-lag correlations calculated by Spearman rank correlation coefficients were employed to assess whether correlations between new COVID-19 cases and internet searches were affected by time. We also constructed a prediction model of new COVID-19 cases using the number of COVID-19 cases, tests, and GT and NAVER RSVs in lag periods (of 1-3 days). Single and multiple regressions were employed using backward elimination and a variance inflation factor of <5.

**Results:**

The numbers of COVID-19–related queries in South Korea increased during local events including local transmission, approval of coronavirus test kits, implementation of coronavirus drive-through tests, a face mask shortage, and a widespread campaign for social distancing as well as during international events such as the announcement of a Public Health Emergency of International Concern by the World Health Organization. Online queries were also stronger in women (*r*=0.763-0.823; *P*<.001) and age groups ≤29 years (*r*=0.726-0.821; *P*<.001), 30-44 years (*r*=0.701-0.826; *P*<.001), and ≥50 years (*r*=0.706-0.725; *P*<.001). In terms of spatial distribution, internet search data were higher in affected areas. Moreover, greater correlations were found in mobile searches (*r*=0.704-0.804; *P*<.001) compared to those of desktop searches (*r*=0.705-0.717; *P*<.001), indicating changing behaviors in searching for online health information during the outbreak. These varied internet searches related to COVID-19 represented community health risk perceptions. In addition, as a country with a high number of coronavirus tests, results showed that adults perceived coronavirus test–related information as being more important than disease-related knowledge. Meanwhile, younger, and older age groups had different perceptions. Moreover, NAVER RSVs can potentially be used for health risk perception assessments and disease predictions. Adding COVID-19–related searches provided by NAVER could increase the performance of the model compared to that of the COVID-19 case–based model and potentially be used to predict epidemic curves.

**Conclusions:**

The use of both GT and NAVER RSVs to explore patterns of community health risk perceptions could be beneficial for targeting risk communication from several perspectives, including time, population characteristics, and location.

## Introduction

The World Health Organization (WHO) declared the COVID-19 outbreak a pandemic on March 11, 2020 [1]. By May 31, 2020, the disease had infected 5,934,936 individuals worldwide [2] including 11,468 individuals in South Korea. The first COVID-19 case in South Korea was confirmed on January 20, 2020 [3]. Slow upturns in disease transmission were reported before February 19, 2020; the local clusters observed in Daegu led to daily increases in the number of new cases [4]. Numerous approaches were undertaken to prevent disease transmission, including coronavirus drive-through testing and social distancing [5,6]. Coronavirus drive-through tests were identified as a safe and efficient screening approach, with each test taking approximately 10 minutes, thus minimizing cross-infection among people being tested [6]. To date, the average number of daily new cases is lower by ten-fold or more compared to those during the peak of the epidemic (from February 19 to March 15, 2020) [3]. Consequently, South Korea is considered among the best-performing countries in tackling the pandemic.

On the contrary, adequate risk communication could also have helped minimize the impacts of disease transmission [7]. Thus, in the pandemic period, the WHO suggests regular risk communication by updating the public and stakeholders on any changes in the status of the pandemic [8]. This action might be challenging because proper risk communication needs a robust understanding of risk perceptions, which helps to identify what knowledge the public needs [7]. However, studies exploring risk perception are often conducted using survey methods or content analyses [7,9-11], which require more resources and longer time. In particular, when investigating an emerging disease, those approaches might be less affordable since the health system will be overburdened with the surge of health care use, thus resulting in more barriers to assessing community health risk perceptions.

Therefore, this study aims to explore patterns of community health risk perceptions toward COVID-19 in South Korea using internet search data. This study is part of infodemiological research that was first introduced in 1996 [12] and explores the distribution of information on the internet [13] for public health and policy about the ground situation in the population. Infodemiology commonly deals with disease-related topics as well as outbreaks and epidemics [14]. This approach can potentially be used since internet query data can be provided easily, promptly, [15], and in a cost-effective manner compared to survey methods [16], and it can potentially capture anomalous patterns in near real time [17].

In this analysis, we used COVID-19–related internet search data provided by Google Trends (GT) and NAVER to represent online queries from the world’s largest search engine and Korean local search engine, which has a higher market share than Google in South Korea [18]. This study explores patterns of public health risk perceptions toward the ongoing outbreak from several different perspectives, including time, population characteristics, and location as used in epidemiological studies. We also constructed a prediction model of new COVID-19 cases using the number of COVID-19 cases, tests, and GT and NAVER relative search volumes (RSVs) in lag periods (of 1-3 days). Future studies are warranted to define the best lag period to perform effective risk communication in the early stages of a disease outbreak.

## Methods

### Data Sets

The daily numbers of new COVID-19 cases and coronavirus tests from January 20 to May 31, 2020, were collected from the Kaggle open-access data set by Kim and colleagues [3]. We used the Time.csv data set to retrieve the number of new daily COVID-19 cases and daily tests, and the TimeProvince.csv data set to collect cumulative coronavirus cases by region. Those data sets covered all cities in South Korea. In addition, internet search data related to COVID-19 were retrieved from the GT [19] and NAVER websites [20] in the same collocation. The information searched was collected 6 weeks earlier from December 5, 2019, to explore patterns before the occurrence of the first COVID-19 case in South Korea. Data were collected using COVID-19–related terms, including coronavirus (코로나 바이러스), coronavirus test (코로나 바이러스 테스트), Middle East respiratory syndrome (MERS; 메르 스), face mask (마스크), social distancing (사회적 거리두기), and Shinchoenji (신천지) in the Korean language, and data were retrieved according to time, gender, age groups, types of device, and location. These keywords were used to represent online information searches for COVID-19–related information, personal protective measures, and preventive approaches. Specific keywords for MERS (메르스) were used to assess whether there was an increase of information searches in the early stage of the outbreak using specific terms related to MERS as reported in previous research [21]. In addition, the Shinchoenji (신천지) keyword was also used to collect online information searches following a cluster in the Shinchoenji church and to define whether this cluster induced a surge of online information searches. For terms that were more than one word, quotes were used to increase the accuracy of data in both GT and NAVER as suggested in an earlier GT research framework [22]. The health category and web search option for GT queries were also used.

Online search data retrieved from GT and NAVER are presented as a relative number called the RSVs that ranges from 0 to 100. The RSVs represent search requests made to those search engines. For GT, the RSVs for a specific term are normalized according to the corresponding time and location [23]. GT RSVs can be downloaded for different times and locations [19], while NAVER provides queries for various times, genders, ages, and types of device categories [20].

### Statistical Analysis

Analyses of health risk perceptions toward COVID-19 were performed using data from January 20 to March 22, 2020. This time frame was selected since this study aims to explore patterns of internet searches representing health risk perceptions in the initial weeks of the outbreak. Data were analyzed in a single graphical form to explore trends in new COVID-19 cases, numbers of tests, and internet searches on a daily basis. Time-lag correlations calculated by Spearman rank correlation coefficients were employed to assess whether correlations of new COVID-19 cases with GT and NAVER RSVs were affected by time within 3 days of a lag or lead period. Statistical analyses were performed using Stata 13 (StataCorp), and strong correlations were defined as correlation coefficients *r*>0.7. Moreover, multilayer maps created using Tableau Public 2020 (Tableau Software, Inc) were generated to define the distributions of new COVID-19 cases and internet searches.

This study also undertakes the task of predicting new COVID-19 cases. Several predictors, including the number of COVID-19 cases, tests, and GT and NAVER RSVs in lag periods (of 1-3 days) were used to predict the target variable, which was the number of new COVID-19 cases. The prediction value was calculated using single and multiple linear regressions employing backward elimination and a variance inflation factor (VIF) of <5 in Stata 13. A lower VIF level was considered to minimize the presence of multicollinearity in the model, particularly in epidemiologic studies [24]. Models were constructed using the development data set (January 20 to March 22, 2020) as used in health risk perception analyses and validated using the future validation data set (March 23 to May 31, 2020). The root mean squared error (RMSE) was assessed for evaluating the models’ performances, as well as Akaike information criterion (AIC) for selecting a correct model and Bayesian information criterion (BIC) for finding the best model for future predictions [25].

## Results

Community health risk perceptions captured by GT and NAVER RSVs were divided into several parts including patterns by time, population characteristics, and location.

### Trends in New COVID-19 Cases, Number of Tests, and Internet Searches on a Daily Basis

**Figure 1:**
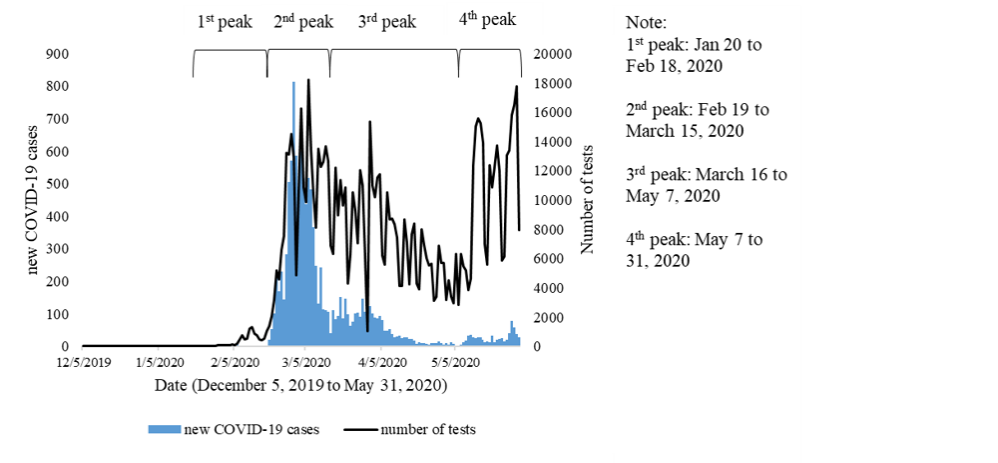
Time series of new COVID-19 cases and number of tests in South Korea.

South Korea reported the first case of COVID-19 on January 20, 2020 (Figure 1), with four peaks of disease transmissions as of May 31, 2020. The first peak occurred until February 18, 2020. The average new cases increased to 311 per day and decreased to 50 cases per day since March 16, 2020. The fourth peak was observed on May 8, 2020, which corresponded with implementation of a new normal starting on May 6, 2020 [26]. Furthermore, as of May 31, 2020, South Korea had reported 11,468 cases of COVID-19. Large numbers of tests were also performed during the outbreak. South Korea performed 6848 tests on average per day from January 20 to May 31, 2020, and 910,822 tests in total, making South Korea one of the countries with the highest number of tests performed.

**Figure 2:**
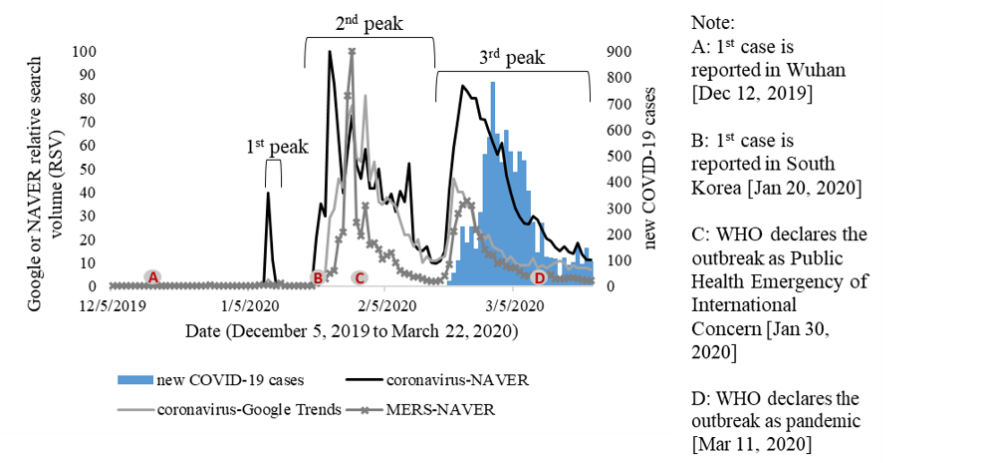
Time series of new COVID-19 cases and Google Trends and NAVER relative search volumes related to the coronavirus and MERS in South Korea. MERS: Middle East respiratory syndrome; WHO: World Health Organization.

During the outbreak, trends of information searches for coronavirus (코로나 바이러스) captured by GT and NAVER were similar (Figure 2). Three peaks of internet searches were observed in the second and fifth weeks of January and in the fourth week of February 2020. Coronavirus-related searches remained high for several days after the first COVID-19 case was reported in Wuhan on December 12, 2019, along with MERS (메르 스)–related queries, which were also elevated in the last two peaks. However, massive surges of information searches occurred along with the identification of the first COVID-19 case in South Korea on January 20 and with the WHO’s declaration of the Public Health Emergency of International Concern (PHEIC) on January 30, 2020. Compared to the daily data on new COVID-19 cases, information searches provided by GT and NAVER peaked 7-9 days earlier. The third peak of coronavirus searches possibly corresponded to the immense increase in the number of new COVID-19 cases due to local transmission. Searches gradually decreased even after the outbreak was declared a pandemic by the WHO on March 11, 2020 [1].

**Figure 3:**
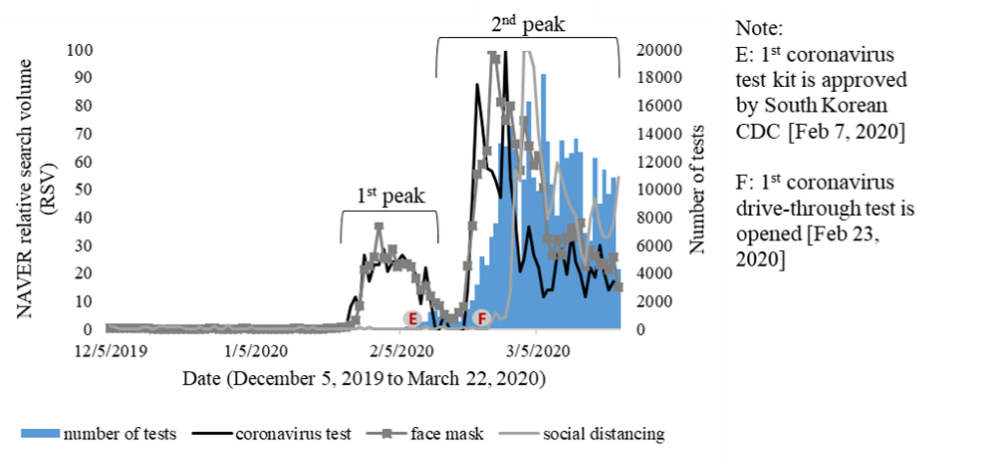
Time series of the daily number of coronavirus tests and NAVER relative search volumes related to coronavirus tests, face masks, and social distancing in South Korea. CDC: Centers for Disease Control and Prevention.

Furthermore, coronavirus test–related (코로나 바이러스 테스트) searches were not captured in GT; hence, Figure 3 only illustrates NAVER RSVs related to coronavirus tests, face masks, and social distancing. Increases in internet searches were observed weeks after the COVID-19 cases were reported and before a coronavirus test kit was approved on February 7, 2020 [27]. The second wave of information searches was found in the third week of February 2020, which might have been caused by an increase in the number of new COVID-19 cases and the implementation of coronavirus drive-through tests on February 23, 2020 [6]. However, patterns of coronavirus test–related searches seemed more similar to trends of new COVID-19 cases compared to the daily numbers of tests.

**Figure 4:**
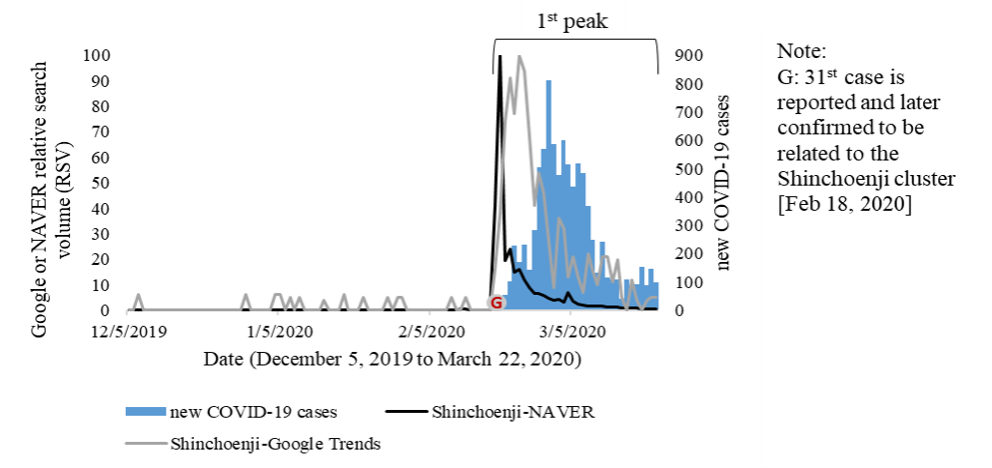
Time series of new COVID-19 cases, Google Trends, and NAVER relative search volumes related to the Shinchoenji cluster in South Korea.

Similar patterns of online queries about coronavirus tests were also identified for face masks (마스크). From the perspective of personal protective measures, the number of face mask–related queries increased in the same period when people began to search for coronavirus tests and face mask shortages in early February [28] and gradually declined in late February, as a regular supply of face masks was provided by the federal government [29]. Moreover, the massive increase in locally acquired cases also induced internet searches related to social distancing (사회적 거리두기) as one of the preventive approaches. Those searches reached a peak as a widespread campaign for social distancing was commended in the first week of March 2020 in South Korea [5]. In contrast, the number of Shinchoenji (신천지)–related searches increased as the Shinchoenji cluster was discovered on February 18, 2020 [30], and gradually decreased thereafter, even before the surge in new COVID-19 cases peaked on February 29, 2020 (Figure 4).

### Time-Lag Correlations Between new COVID-19 Cases and Internet Searches in Different Gender and age Groups

The results in Tables 1 and 2 demonstrated a moderate correlation (*r*=0.628) between new COVID-19 cases and GT RSVs related to coronavirus with a lag of 3 days. On the contrary, a strong correlation (*r*=0.718) of coronavirus information searches counting for both men and women with a lag of 3 days showed no differences for NAVER RSVs. However, the correlations varied across different age groups and lag periods. Strong correlations were observed with a lag of 3 days for all ages (*r*=0.729) and those aged ≤18 years (*r*=0.821), 19-24 years (*r*=0.784), 25-29 years (*r*=0.726), 50-54 years (*r*=0.706), and ≥50 years (*r*=0.725). Meanwhile, the weakest correlation was found in the age group of 35-39 years (*r*=0.622). The ≤18 years and 19-24 years age groups for NAVER RSVs had strong correlations in almost all lag and lead periods. Moreover, the strength of the correlations decreased in the lead period or a few days after the number of new COVID-19 cases increased for both GT and NAVER RSVs. Compared to NAVER RSVs, GT RSVs for coronavirus had weaker correlations with new COVID-19 cases.

**Table 1:** Time-lag correlation coefficients between new COVID-19 cases, Google Trends, and NAVER relative search volumes related to the coronavirus in South Korea.

Day		Google Trends	NAVER												
			Gender			Age groups (years)									
			Men	Women	Overall		≤18	19-24	25-29	30-34	35-39	40-44	45-49	50-54	≥55
–**3 days**															
	*r*	0.628^a^	*0.718* ^a,b^	*0.718* ^a^	*0.729* ^a^		*0.821* ^a^	*0.784* ^a^	*0.726* ^a^	0.661^a^	0.622^a^	0.648^a^	0.685^a^	*0.706* ^a^	*0.725* ^a^
	*P* value	<.001	<.001	<.001	<.001		<.001	<.001	<.001	<.001	<.001	<.001	<.001	<.001	<.001
–**2 days**															
	*r*	0.605	0.684	0.684	0.694		*0.805*	*0.759*	0.696	0.621	0.581	0.607	0.655	0.680	0.693
	*P* value	<.001	<.001	<.001	<.001		<.001	<.001	<.001	<.001	<.001	<.001	<.001	<.001	<.001
–**1 day**															
	*r*	0.590	0.670	0.670	0.681		*0.812*	*0.759*	0.678	0.601	0.561	0.593	0.638	0.662	0.682
	*P* value	<.001	<.001	<.001	<.001		<.001	<.001	<.001	<.001	<.001	<.001	<.001	<.001	<.001
**0 days**															
	*r*	0.576	0.654	0.654	0.663		*0.803*	*0.737*	0.659	0.578	0.538	0.565	0.606	0.634	0.655
	*P* value	<.001	<.001	<.001	<.001		<.001	<.001	<.001	<.001	<.001	<.001	<.001	<.001	<.001
**1 day**															
	*r*	0.554	0.647	0.647	0.661		*0.794*	*0.736*	0.660	0.579	0.536	0.560	0.606	0.633	0.658
	*P* value	<.001	<.001	<.001	<.001		<.001	<.001	<.001	<.001	<.001	<.001	<.001	<.001	<.001
**2 days**															
	*r*	0.505	0.591	0.591	0.606		*0.759*	0.688	0.600	0.513	0.477	0.508	0.554	0.580	0.606
	*P* value	<.001	<.001	<.001	<.001		<.001	<.001	<.001	<.001	<.001	<.001	<.001	<.001	<.001
**3 days**															
	*r*	0.491	0.579	0.579	*0.597*		*0.749*	0.682	0.587	0.500	0.468	0.498	0.537	0.565	0.592
	*P* value	<.001	<.001	<.001	<.001		<.001	<.001	<.001	<.001	<.001	<.001	<.001	<.001	<.001

^a^Strongest correlation for each column.

^b^Italics represent a strong correlation with *r*>0.7.

**Table 2:** Time-lag correlation coefficients between new COVID-19 cases, Google Trends, and NAVER relative search volumes related to the coronavirus test in South Korea.

Day		Google Trends	NAVER											
			Gender		Age groups (years)									
			Men	Women	Overall	≤18	19-24	25-29	30-34	35-39	40-44	45-49	50-54	≥55
–**3 days**														
	*r*	N/A^a^	*0.739* ^b^	*0.769*	*0.770*	0.595^c^	0.681	0.654	*0.701*	*0.734*	0.696	0.624	0.612^c^	0.441
	*P* value		<.001	<.001	<.001	<.001	<.001	<.001	<.001	<.001	<.001	<.001	<.001	<.001
–**2 days**														
	*r*	N/A	*0.769*	*0.790*	*0.797*	0.505	0.650	0.687	*0.752*	*0.786*	0.692	0.673^c^	0.581	0.445
	*P* value		<.001	<.001	<.001	<.001	<.001	<.001	<.001	<.001	<.001	<.001	<.001	<.001
–**1 day**														
	*r*	N/A	*0.795* ^b,c^	*0.799*	*0.824*	0.500	*0.725* ^c^	0.645	*0.775*	*0.826* ^c^	*0.704*	0.630	0.532	0.434
	*P* value		<.001	<.001	<.001	<.001	<.001	<.001	<.001	<.001	<.001	<.001	<.001	<.001
**0 days**														
	*r*	N/A	*0.778*	*0.799*	*0.812*	0.542	*0.720*	0.653	*0.746*	*0.783*	*0.755* ^c^	0.559	0.551	0.358
	*P* value		<.001	<.001	<.001	<.001	<.001	<.001	<.001	<.001	<.001	<.001	<.001	<.001
**1 day**														
	*r*	N/A	*0.775*	*0.823* ^c^	*0.828* ^c^	0.508	0.682	0.688^c^	*0.786* ^c^	*0.814*	*0.718*	0.586	0.557	0.450^c^
	*P* value		<.001	<.001	<.001	<.001	<.001	<.001	<.001	<.001	<.001	<.001	<.001	<.001
**2 days**														
	*r*	N/A	*0.756*	*0.802*	*0.805*	0.549	0.620	0.623	*0.774*	*0.762*	*0.731*	0.586	0.537	0.433
	*P* value		<.001	<.001	<.001	<.001	<.001	<.001	<.001	<.001	<.001	<.001	<.001	<.001
**3 days**														
	*r*	N/A	*0.744*	*0.763*	*0.781*	0.465	0.572	0.606	0.694	*0.756*	0.633	0.633	0.518	0.424
	*P* value		<.001	<.001	<.001	<.001	<.001	<.001	<.001	<.001	<.001	<.001	<.001	<.001

^a^N/A: not applicable.

^b^Italics represent strong correlations with r>0.7.

^c^Strongest correlation for each column.

Different patterns were noted in coronavirus test–related searches. No correlation could be calculated for GT RSVs due to the insufficient number of queries recorded. Strong correlations were found with a lag of 1 day for men (*r*=0.795) and a lead of 1 day for women (*r*=0.823) for NAVER RSVs, as well as for all age groups with a lead of 1 day (*r*=0.828). Moreover, weak to strong correlations were reported in different age groups. The 19-24 years age group had a strong correlation (*r*=0.725) with a lag of 1 day, followed by the 30-34 years age group (*r*=0.786 with a lead of 1 day), 35-39 years age group (*r*=0.826 with a lag of 1 day), and 40-44 years age group (*r*=0.755 with a lag of 0 days).

### Trends in Online Information Searches Based on the Type of Device Used for Accessing the Internet

**Figure 5:**
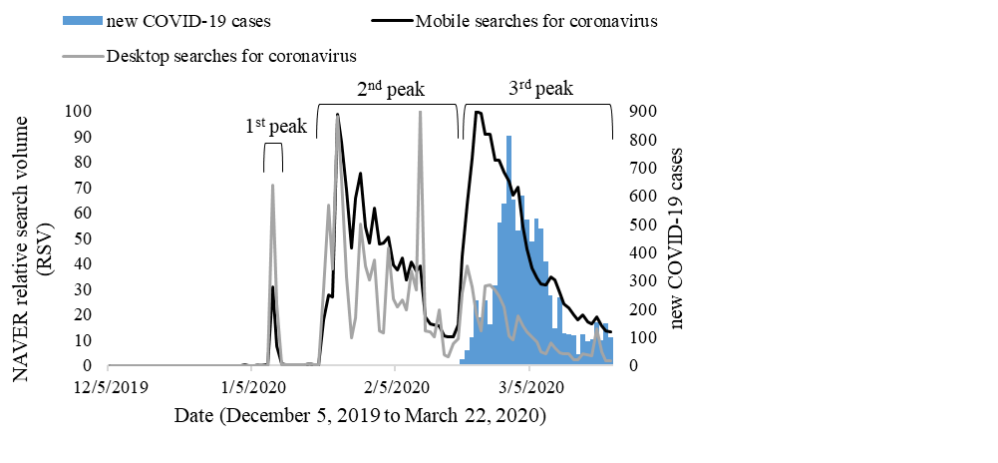
Time series of new COVID-19 cases and NAVER relative search volumes related to the coronavirus in South Korea.

**Figure 6:**
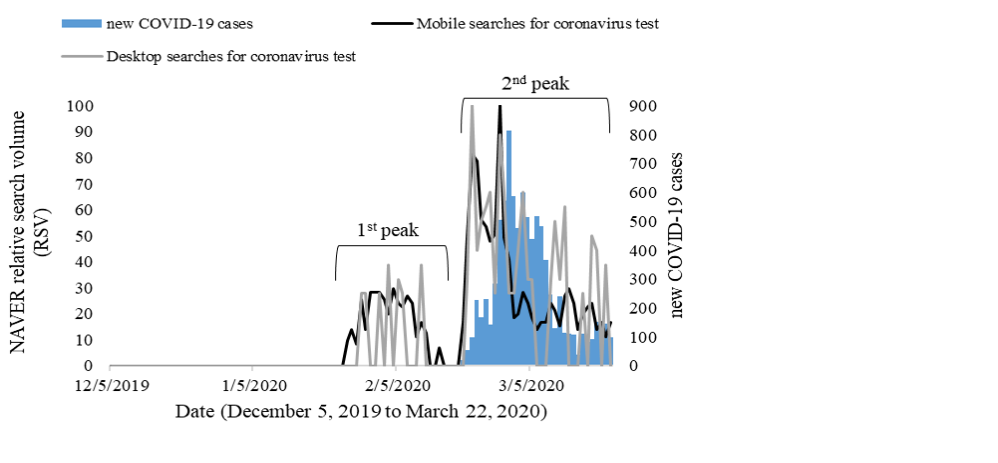
Time series of new COVID-19 cases and NAVER relative search volumes related to the coronavirus test in South Korea.

Figures 5 and 6 show trends of online information searches for coronavirus and coronavirus tests using mobile devices and desktops. Mobile search queries for coronavirus were higher in all peaks of information searches. For coronavirus test–related searches, mobile searches seemed to be more frequent and stable than those of desktop searches in all peaks.

Spearman rank correlation coefficients in Table 3 demonstrated strong correlations for the overall data set (mobile and desktop searches) of coronavirus searches with a lag of 3 days (*r*=0.729), as well as mobile searches (*r*=0.761). Interestingly, mobile searches had stronger correlation coefficients for all lag and lead periods than did overall searches. However, weak to moderate correlations (*r*=0.417-0.546) were observed for coronavirus-related searches through desktop devices. For coronavirus test online searches, strong correlations (*r*=0.770-0.828) were reported for all lag and lead days. Still, mobile searches were observed to have a stronger correlation coefficient than desktop searches. The strongest correlations were found with a lag of 0 days for mobile searches (*r*=0.804) and with a lag of 1 day for desktop searches (*r*=0.717).

**Table 3:** Time-lag correlation coefficients between new COVID-19 cases and NAVER relative search volumes related to the coronavirus and coronavirus test in South Korea.

Day			Coronavirus searches (type of device)						Coronavirus test searches (type of device)				
			Overall		Mobile		Desktop		Overall		Mobile		Desktop
–**3 days**													
	*r*	*0.729* ^a,b^		*0.761* ^a^		0.546^a^		*0.770*		*0.756*		0.677	
	*P* value	<.001		<.001		<.001		<.001		<.001		<.001	
–**2 days**													
	*r*	0.694		*0.726*		0.534		*0.797*		*0.787*		0.657	
	*P* value	<.001		<.001		<.001		<.001		<.001		<.001	
–**1 day**													
	*r*	0.681		*0.720*		0.497		*0.824*		*0.799*		*0.717* ^a^	
	*P* value	<.001		<.001		<.001		<.001		<.001		<.001	
**0 days**													
	*r*	0.663		*0.704*		0.461		*0.812*		*0.804* ^a^		0.638	
	*P* value	<.001		<.001		<.001		<.001		<.001		<.001	
**1 day**													
	*r*	0.661		0.692		0.475		*0.828* ^a^		*0.804*		*0.705*	
	*P* value	<.001		<.001		<.001		<.001		<.001		<.001	
**2 days**													
	*r*	0.606		0.650		0.417		*0.805*		*0.788*		0.654	
	*P* value	<.001		<.001		<.001		<.001		<.001		<.001	
**3 days**													
	*r*	0.597		0.633		0.423		*0.781*		*0.761*		0.626	
	*P* value	<.001		<.001		<.001		<.001		<.001		<.001	

^a^Strongest correlation for each column.

^b^Italics represent a strong correlation with *r*>0.7.

### Distributions of new COVID-19 Cases and Internet Searches

**Figure 7:**
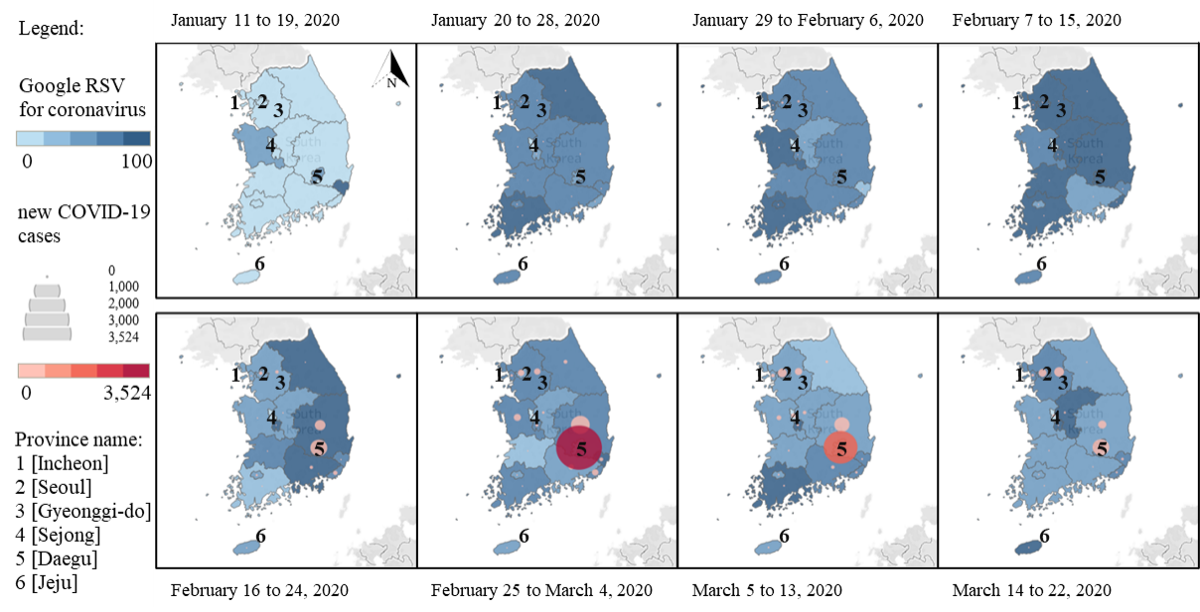
Distribution of new COVID-19 cases and Google Trends RSVs in South Korea. RSV: relative search volume.

Spatial distributions of new COVID-19 cases and GT RSVs are illustrated in Figure 7. Results showed that 9 days before confirmed cases were reported in South Korea, the numbers of GT RSVs related to the coronavirus captured in Gyeonggi-do, Seoul, Chungcheongnam-do, Daegu, and Ulsan Provinces increased. Thereafter, the aforementioned provinces reported COVID-19–confirmed cases. During the early weeks of disease transmission (as of February 15, 2020), COVID-19 had spread in Seoul, Incheon, Gwangju, Gyeonggi-do, and Jeollabuk-do (Figure 7). Similar patterns were also captured for GT RSVs, which seemed to be elevated in those periods in the western part of South Korea where confirmed cases were reported.

Furthermore, a surge in new COVID-19 cases began on February 19, 2020. GT RSVs gradually increased during that period in the eastern part of South Korea, including Daegu, the epicenter of local transmission. Daegu contributed 71.79% of confirmed cases or 262.14 cases per 100,000 population as of March 22, 2020 [31], and had a higher estimated death rate than the national rate [32]. Interestingly, increases in the number of online searches were observed a week before those massively expanding cases in provinces surrounding Daegu. The large numbers of locally acquired cases were reported from February 25 to March 4, 2020, and swiftly declined in mid-March. When the number of new cases decreased, the number of internet searches in the western part of South Korea began to increase, which indicated an elevation in the number of COVID-19 cases in the latter part of the study period.

### Predicting new COVID-19 Cases

Three different models for predicting new COVID-19 cases were established in this study (Table 4). New COVID-19 cases with a lag of 1 day, number of COVID-19 tests with lags of 2 days and 1 day, GT coronavirus searches with a lag of 1 day, and NAVER coronavirus searches with a lag of 3 days were selected as important predictors for the models. Model 1 showed high performance, which indicates that this model represented 89% of new COVID-19 cases in contrast with model 2, which only represented 35% of cases as shown in the adjusted *r*^2^ values. By combining those two models (a case-based model and internet search data–based model), the model’s performance seemed to have slightly increased to nearly 90%, resulting in the lowest RMSE as observed in model 3.

**Table 4:** Prediction model of new COVID-19 cases in South Korea.

Models and predictors		Coef^a^ (95% CI)	*P* value for *F* test	Adjusted *r*^2^	RMSE^b^	AIC^c^	BIC^d^
**Model 1 (predictors included new COVID-19 cases and number of COVID-19 tests)**			<.001	0.891	54.348	1851.326	1864.03
	New COVID-19 cases lag 1 day	0.942 (0.883 to 1.001)					
	Number of tests lag 2 days	–0.004 (–0.007 to –0.001)					
	Number of tests lag 1 day	0.004 (0.001 to 0.007)					
	Cons^e^	3.957 (–5.415 to 13.329)					
**Model 2 (predictors included GT^f^ and NAVER RSVs^g^ related to coronavirus)**			<.001	0.354	133.802	2153.293	2162.805
	GT RSVs lag 1 day	–0.964 (–1.604 to –0.324)					
	NAVER RSVs lag 3 days	3.583 (2.859 to 4.308)					
	Cons	28.920 (4.338 to 53.503)					
**Model 3 (predictors included new COVID-19 cases, number of tests, and GT and NAVER RSVs related to coronavirus)**			<.001	0.895	53.177	1835.169	1851.022
	New COVID-19 cases lag 1 day	0.880 (0.809 to 0.951)					
	Number of tests lag 2 days	–0.004 (–0.006 to –0.001)					
	Number of tests lag 1 day	0.004 (0.002 to 0.007)					
	NAVER RSVs lag 3 days	0.536 (0.177 to 0.894)					
	Cons	–4.334 (–15.136 to 6.467)					

^a^Coef: coefficient.

^b^RMSE: root mean squared error.

^c^AIC: Akaike information criterion.

^d^BIC: Bayesian information criterion.

^e^Cons: constant.

^f^GT: Google Trends.

^g^RSV: relative search volume.

**Figure 8:**
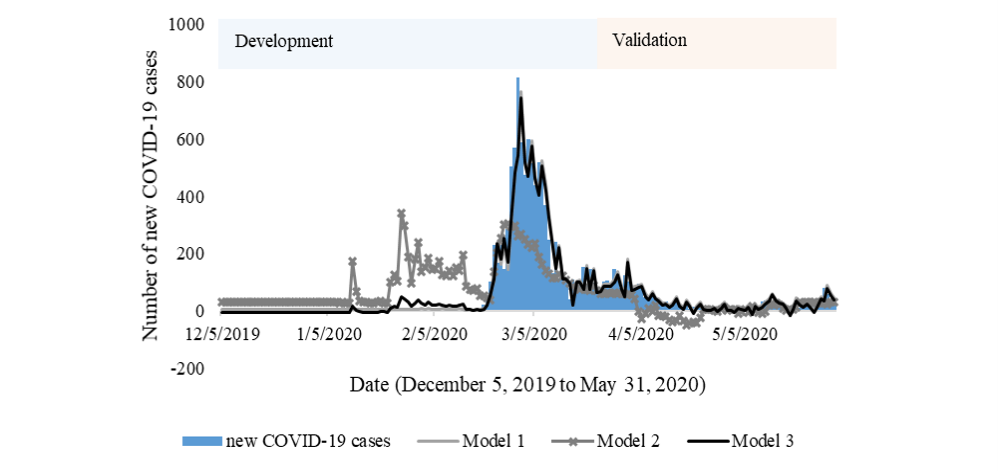
Prediction of new COVID-19 cases in South Korea.

Models were then plotted in Figure 8 for both the development and validation sets. Model 3 performed better compared to the two other models in the development set as assessed by the value of the adjusted *r*^2^ as well as RMSE, AIC, and BIC. In the validation set, this model also performed well, and this was indicated by the RMSE decreasing to 18.320.

## Discussion

### Public Health Risk Perceptions

Risk perception is defined as a person’s subjective judgment toward the likelihood of negative occurrences including diseases or illnesses [33]. In terms of disease outbreaks, understanding community health risk perceptions are needed in the early phase of an outbreak, particularly in the case of an emerging disease. This is because in the initial period, there will be limited treatments, few resources, and delays in active interventions [34]. Therefore, exploring the perception of risk is a necessary step in managing the risk of an outbreak. Since a robust public risk perception assessment could help in divining effective risk communication, this step should be taken immediately to reduce the impact of the COVID-19 outbreak. Consequently, it is more affordable to conduct the community health risk perception assessment using internet search data, since it can be provided more easily, promptly, and cost-effectively compared to survey methods [16] and can potentially capture anomalous patterns in real time [17]. With the widespread use of the internet and mobile devices, internet search data can be more accurate in representing the community health risk perceptions [35], as information-seeking intentions are directly affected by risk perceptions [9].

### Principal Results

In this study, we found various correlations, which ranged from weak to strong, among GT and NAVER RSVs, new COVID-19 cases, and the number of tests. Previous studies also reported strong correlations between GT and NAVER RSVs compared to surveillance data [16,36]. Therefore, increased searches for COVID-19–related information might represent community health risk perceptions during local and international events. NAVER RSVs, as a local search engine that has the largest market share in South Korea (57.31% for all search categories in 2020 as of June 14) [18], seemed to be more sensitive to local issues such as coronavirus tests as shown in Figure 3. A similar result was also reported in a previous study that demonstrated that Baidu (in China) has better predictive performance for disease prediction than GT RSVs [36]. These findings suggest that NAVER RSVs could also potentially complement the use of GT RSVs, which are excessively used in the fields of infodemiology.

Patterns of community risk perceptions retrieved from information searches in this analysis were explained by examining different aspects: time, gender, age groups, types of device used for accessing the internet, and spatial distributions. Patterns according to time revealed that the number of online queries related to COVID-19 increased during local events including local transmission, approval of coronavirus test kits, implementation of coronavirus drive-through tests, a face mask shortage, a widespread campaign for social distancing, and transmission of the Shinchoenji cluster, as well as during international events such as the announcement of the PHEIC. Yet, South Korea was also one of the countries affected by the MERS epidemic [37]. That experience might have also contributed to the increased number of searches for coronavirus information even though cases had not yet been detected until then. Moreover, MERS-related searches also remained high during the study period. These findings indicated that public health risk perceptions increased following both local and international crises. Hence, risk communication should promptly be conducted, considering that health risk perceptions might change over time as the outbreak progresses.

Patterns according to time also revealed decreased numbers of GT and NAVER RSVs in the middle of the epidemic curve, which might have been caused by the extensive availability of online news and health expert reports during that period [38]. It might also have been provoked by decreased risk perceptions as the epidemic progressed [7]. Thus, using internet query data to analyze community risk perceptions could be useful in the early stage of an outbreak.

Moreover, patterns categorized by different age groups revealed that younger (≤29 years) and older age groups (≥50 years) had strong correlations of internet searches for coronavirus information with new COVID-19 cases. This finding demonstrated the high-risk perceptions of those age groups, even 3 days before an increase in the number of new COVID-19 cases locally. High-risk perceptions in younger age groups might have been induced by massive internet access for acquiring information and high numbers of confirmed cases in that age group (33.24%) in South Korea [31,39]. Meanwhile, perceived vulnerability might be common in older age groups, since an older age is one of the prominent risk factors for COVID-19 mortality [40], and 98.08% of fatal cases in South Korea occurred in older adults [31]. Additionally, a previous study showed that the older age group had higher risk perceptions [7].

In contrast, the age group of 30-49 years only showed weak to moderate correlations even 3 days before the event. This might have been due to the lower percentage of confirmed cases (23.94%) in that age group compared to that in the younger age group (≤29 years), which could also have influenced health risk perceptions. Meanwhile, online queries concerning coronavirus tests showed high-risk perceptions in the 35-44 years age group. These findings illustrate that adults perceived the coronavirus test–related information to be more important than disease-related knowledge. It might also have been influenced by the massive number of coronavirus tests conducted so far. Meanwhile, younger (aged ≤29 years) and older age groups (aged ≥50 years) had a different perception, thereby making infection-related information an essential search. In terms of gender, both men and women perceived the coronavirus as having similar levels of risk, but risk perception for coronavirus tests was higher among women. This result is similar to that reported in a previous study, which showed a higher risk perception in the women’s group [7]. Hence, health risk communication should target both men and women as well as vulnerable age groups.

As to device use, patterns demonstrated that mobile device searches had stronger correlations with COVID-19–related searches compared to desktop queries. Strong correlations for mobile device searches were even observed 3 days before the outbreak. However, desktop searches showed a strong correlation with a lag of 1 day, which was 2 days later compared to mobile searches. This finding implies that high-risk perceptions stimulated an enormous number of mobile searches during the outbreak period. Identical results were also illustrated in a previous study by Shin and colleagues [16]. The widespread use of mobile devices in the digital era [35] has promoted changes in behavior from desktop to mobile device users. Therefore, the government should ensure that risk communication can be easily accessed through mobile platforms for rapid dissemination. Research findings also demonstrated that the spatial distributions of internet searches were higher in locations with new COVID-19 cases. This finding was similar to that in previous studies, which indicated that individuals in affected areas have higher risk perceptions [7,11].

Later in the analysis, we also addressed the prediction of new COVID-19 cases using three different models. Results showed that adding COVID-19–related searches provided by NAVER could increase the performance of the model compared to that of the COVID-19 case–based model. This result resembled an earlier study [17], which also found that a model’s performance increased with use of internet search data from local search engines. Furthermore, in the validation set, this model performed better, which might have been caused by a longer period for querying NAVER data; therefore, trends could be adjusted better and affect the model’s performance in the validation set. Hence, considering NAVER RSVs data for case prediction could be important, employing the same data set to better understand health risk perceptions is also of importance, particularly in the early stage of an outbreak.

Briefly, this study provides a depiction of community health risk perceptions toward COVID-19 in South Korea, which tended to be higher in the period of local and international events, also for women, certain age groups, and people in affected areas. During the outbreak, people were more likely to access the internet through mobile devices, which are potential channels where health risk communication can be effectively and densely disseminated. Moreover, NAVER RSVs can potentially be used for health risk perception assessments and disease prediction. This method demonstrated an easy and low-cost approach for estimating health risk perceptions during a pandemic. Since providing a rapid risk perception assessment is needed in the early stage of an outbreak, combining GT and NAVER RSVs could be beneficial for targeting risk communication in terms of time, population characteristics, and location. GT RSVs alone only revealed patterns according to time and location [41]. However, this study only explored the positive risk perceptions toward COVID-19 rather than negative risk perceptions such as psychological impacts. As multiple studies also reported increases in incidence of anxiety, depression, anger, insomnia, distress, and suicidality during the initial phase of the epidemic [42], exploring the negative risk perceptions of the COVID-19 pandemic would be important for future works.

### Limitations

As online search queries might change over time, identifying the best lag time for conducting risk communication is challenging. However, using either GT or NAVER RSVs allowed flexibility in defining the time range of data queries. Thus, we can collect adequate retrospective data sets for identifying the best lag time. In addition, this analysis might be limited to specific time frames and included only two popular search engines and certain keywords, as well as was limited for positive risk perceptions. Therefore, further research that considers those aspects to improve results of the risk perception analysis is required.

### Conclusions

Community health risk perceptions toward the COVID-19 outbreak in South Korea observed from GT and NAVER RSVs increased during local and international events and were higher in women, certain age groups, and in affected areas. Although NAVER RSVs tended to be more sensitive in terms of local issues, integrating GT and NAVER RSVs could potentially provide varied search patterns in terms of time, population characteristics, and location. Moreover, online searches also identified important variables in predicting epidemic curves in the initial stage of an outbreak.

## Abbreviations

AIC: Akaike information criterion
BIC: Bayesian information criterion
GT: Google Trends
MERS: Middle East respiratory syndrome
MOE: Ministry of Education
MOST: Ministry of Science and Technology
NRF: National Research Foundation of Korea
PHEIC: Public Health Emergency of International Concern
RMSE: root mean squared error
RSV: relative search volume
VIF: variance inflation factor
WHO: World Health Organization
